# Increased Central Auditory Gain and Decreased Parvalbumin-Positive Cortical Interneuron Density in the *Df1/+* Mouse Model of Schizophrenia Correlate With Hearing Impairment

**DOI:** 10.1016/j.bpsgos.2022.03.007

**Published:** 2022-03-16

**Authors:** Fhatarah A. Zinnamon, Freya G. Harrison, Sandra S. Wenas, Qing Liu, Kuan Hong Wang, Jennifer F. Linden

**Affiliations:** a Ear Institute, University College London, London, United Kingdom; b Department of Neuroscience, Physiology & Pharmacology, University College London, London, United Kingdom; c Unit on Neural Circuits and Adaptive Behaviors, Clinical and Translational Neuroscience Branch, National Institute of Mental Health, Bethesda, Maryland; d Department of Neuroscience, Del Monte Institute for Neuroscience, University of Rochester Medical Center, Rochester, New York

**Keywords:** 22q11.2 deletion syndrome, Auditory cortex, Auditory evoked potentials, DiGeorge syndrome, Parvalbumin-positive interneurons, Velocardiofacial syndrome

## Abstract

**Background:**

Hearing impairment is a risk factor for schizophrenia. Patients with 22q11.2 deletion syndrome have a 25% to 30% risk of schizophrenia, and up to 60% also have varying degrees of hearing impairment, primarily from middle-ear inflammation. The *Df1/+* mouse model of 22q11.2 deletion syndrome recapitulates many features of the human syndrome, including schizophrenia-relevant brain abnormalities and high interindividual variation in hearing ability. However, the relationship between brain abnormalities and hearing impairment in *Df1/+* mice has not been examined.

**Methods:**

We measured auditory brainstem responses, cortical auditory evoked potentials, and/or cortical parvalbumin-positive (PV^+^) interneuron density in over 70 adult mice (32 *Df1/+*, 39 wild-type). We also performed longitudinal auditory brainstem response measurements in an additional 20 animals (13 *Df1/+*, 7 wild-type) from 3 weeks of age.

**Results:**

Electrophysiological markers of central auditory excitability were elevated in *Df1/+* mice. PV^+^ interneurons, which are implicated in schizophrenia pathology, were reduced in density in the auditory cortex but not the secondary motor cortex. Both auditory brain abnormalities correlated with hearing impairment, which affected approximately 60% of adult *Df1/+* mice and typically emerged before 6 weeks of age.

**Conclusions:**

In the *Df1/+* mouse model of 22q11.2 deletion syndrome, abnormalities in central auditory excitability and auditory cortical PV^+^ immunoreactivity correlate with hearing impairment. This is the first demonstration of cortical PV^+^ interneuron abnormalities correlating with hearing impairment in a mouse model of either schizophrenia or middle-ear inflammation.

The multigene deletion that causes 22q11.2 deletion syndrome (22q11.2DS) is the strongest known cytogenetic risk factor for schizophrenia in humans (1,2). Approximately 25% to 30% of patients with 22q11.2DS develop schizophrenia during adolescence or adulthood (1,3,4). Up to 60% of patients with 22q11.2DS have hearing impairment (HI), arising primarily from high rates of recurrent or chronic otitis media (middle-ear inflammation) (5).

The *Df1*/+ mouse model of 22q11.2DS has an engineered hemizygous deletion of 1.2 Mbp encompassing 18 orthologs of genes deleted in human 22q11.2DS (6). Similar to other mouse models of 22q11.2DS, the *Df1*/+ mouse recapitulates many phenotypic features of the human syndrome (2,6,7), including brain and behavioral anomalies that have been linked to schizophrenia in humans (8,9). *Df1*/+ mice exhibit reduced prepulse inhibition of the acoustic startle response (10), an auditory behavioral marker for schizophrenia-like abnormalities and a common feature of 22q11.2DS in humans (4). Specific abnormalities in auditory thalamocortical processing have also been reported in *Df1*/+ mice, including abnormal sensitivity of auditory thalamocortical projections to antipsychotic drugs (11). However, similar to humans with 22q11.2DS, up to 60% of *Df1*/+ mice have HI (12), which arises from developmental defects that increase susceptibility to otitis media (13). The potential interaction between HI and auditory brain abnormalities in *Df1*/+ mice has never been systematically explored.

In humans, HI has been described as the “neglected risk factor for psychosis” (14). Hearing loss is associated with increased risk of psychosis and hallucinations, and HI in childhood elevates the risk of developing schizophrenia later in life (15). The mechanisms underlying the association between HI and schizophrenia are unknown and could include common etiology, top-down influences (e.g., from social isolation), and/or bottom-up effects. A role for bottom-up effects is suggested by data from animal studies indicating that reductions in peripheral auditory input drive long-lasting changes in central auditory processing, which can affect behavior (16, 17, 18, 19). Even moderate conductive HI, such as that caused by otitis media, can produce persistent changes in inhibitory synaptic transmission in the auditory cortex that persist after normal hearing is restored (17,18,20,21).

Here, we investigated the relationship between HI and auditory brain abnormalities in the *Df1/+* mouse model of 22q11.2DS. We focused on neurophysiological and neuroanatomical markers associated with schizophrenia in humans, such as abnormalities in cortical auditory evoked potentials (AEPs) (22) and parvalbumin-positive (PV^+^) cortical interneurons (23). PV^+^ interneurons play a key role in maintaining excitation-inhibition balance in the cortex (24), and abnormalities in these inhibitory cells are thought to be important to the pathophysiology of schizophrenia (25). Our results reveal a significant correlation between HI in *Df1/+* mice and both electrophysiological markers of central auditory excitability and reductions in density of PV-expressing cortical interneurons. Thus, interindividual differences in the magnitude of brain abnormalities in the *Df1/+* mouse model of 22q11.2DS can be predicted from interindividual differences in the degree of peripheral HI.

## Methods and Materials

### Animals

*Df1/+* (also known as *Df(16)1/+*) mice (6) and their wild-type (WT) littermates were maintained in standard mouse housing facilities at either University College London or the National Institute of Mental Health. Experiments at University College London were performed in accordance with a Home Office project license approved under the United Kingdom Animal Scientific Procedures Act of 1986. Experiments at the National Institute of Mental Health were approved by the local Animal Care and Use Committee. See the Supplement for further details.

### Neurophysiology

Auditory brainstem response (ABR) and cortical AEP recordings were obtained from mice anesthetized with ketamine and either medetomidine or dexmedetomidine. All testing was performed in a sound isolation booth. Auditory stimuli were presented either via an in-ear coupler (for longitudinal ABR threshold measurements) or free-field from a speaker directed at the ear under test. For ABR threshold measurements, we used click or tone stimuli presented at 0 to 90 dB sound pressure level (SPL). For comparisons of ABR and AEP wave magnitudes and latencies, we used 80-dB SPL click stimuli. ABR signals were recorded differentially between subdermal electrodes placed at the vertex (+) and behind or below the ear being tested (−), with a ground electrode either near the opposite ear (ABR threshold measurements) or over the olfactory bulb (ABR/AEP comparisons). AEP signals were recorded single-ended from subdermal electrodes placed over the temporal lobe contralateral to the ear being tested, relative to a ground over the olfactory bulb. See the Supplement for further details.

### Immunohistochemistry and Microscopy

Coronal brain sections (50 μm thick) were either stained alternately for Nissl substance and PV or triple-stained for PV, NeuN, and DAPI. Sections stained for Nissl or DAPI were viewed at 2.5× to 5× magnifications to identify auditory cortex (A1) and secondary motor cortex (M2) with reference to a mouse brain atlas (26). Single-plane images of A1 and M2 sections immunostained for PV and NeuN were then taken at 5× to 10× magnification with 720 pixels/inch resolution, using a Zeiss Axio Scan 2 Imaging microscope. See the Supplement for further details.

### Data Analysis

#### ABRs and AEPs

ABR signals were bandpass filtered in software (100–3000 Hz, 5th-order Butterworth) before averaging across trials; AEP signals were unfiltered aside from the 2.2- to 7500-Hz hardware filter used in data collection. The ABR threshold was identified as in previous work (12). ABR wave I amplitude was defined as the difference in signal amplitude between the moment of sound onset and the first peak of the ABR to an 80-dB SPL click. AEP P1-N1 and N1-P2 amplitudes were defined as amplitude differences between the respective AEP wave components (27): P1, maximum peak 15 to 30 ms after stimulus onset; N1, maximum negative deflection 25 to 60 ms after onset; and P2, maximum peak 60 to 110 ms after onset. Central auditory gain was defined as the amplitude of the AEP wave complex (P1-N1 or N1-P2) divided by the amplitude of ABR wave I.

#### Cell Counts

PV^+^ and NeuN^+^ cell counts and laminar distributions in A1 and M2 were estimated for images from both hemispheres in each mouse when possible. Immunohistochemical data from some hemispheres and animals were lost due to problems with perfusion, damage to sections, or aberrant fluorescence in images. See Table 1 for the numbers of mice and hemispheres used for comparisons shown in Results figures.

**Table 1:** Numbers of Mice and Brain Hemispheres Used for Analyses of PV^+^ and NeuN^+^ Cell Density and Laminar Distribution

Cell Type	Area	Sample Units	All WT	All *Df1/+*	WT With ABR	*Df1/+* With ABR
PV^+^	A1	Mice	19	19	14	14 (6 NHI, 8 HI)
		Hemispheres	32	34	22	24 (10 NHI, 14 HI)
	M2	Mice	25	21	19	16 (7 NHI, 9 HI)
		Hemispheres	43	36	31	28 (11 NHI, 17 HI)
NeuN^+^	A1	Mice	7	10	7	10 (4 NHI, 6 HI)
		Hemispheres	10	15	10	15 (6 NHI, 9 HI)
	M2	Mice	14	12	14	12 (6 NHI, 6 HI)
		Hemispheres	25	20	25	20 (10 NHI, 10 HI)

All mice were included in comparisons of WT and *Df1*/+ mice (Figure 6B, E; Figure S2B, E; and Figure S4A, C). Only data from mice that underwent ABR testing were included in comparisons of WT animals and *Df1*/+ mice with and without hearing impairment (Figure 6C, F; Figure S2C, F; Figure S3; and Figure S4B, D).

A1, primary auditory cortex; ABR, auditory brainstem response; HI, hearing impairment; M2, secondary motor cortex; NHI, no hearing impairment; PV, parvalbumin; WT, wild-type.

Cortical areas of interest were defined by overlaying the section image with the mouse atlas image (26) for the corresponding coronal location using Adobe Photoshop Elements. Cell centers were marked and centroid coordinates recorded using ImageJ. For PV analysis, all immunolabeled cells in the cortical area of interest (A1 or M2) were counted. For NeuN analysis, immunolabeled cells were counted within a smaller pia-to-white matter rectangular strip through the center of the region of interest (5% of total area). Laminar distributions of cells were estimated using custom MATLAB software (The MathWorks, Inc.), which calculated cell centroid depth along a line perpendicular to the pial surface and white matter, normalized by the pia-to-white matter distance in each section. Laminar distributions were compared between animal groups using cell counts binned into 5 equal-depth bins by cortical depth; similar analyses were also performed using 10 or 20 bins in depth.

### Statistical Methods

**Figure 1:**
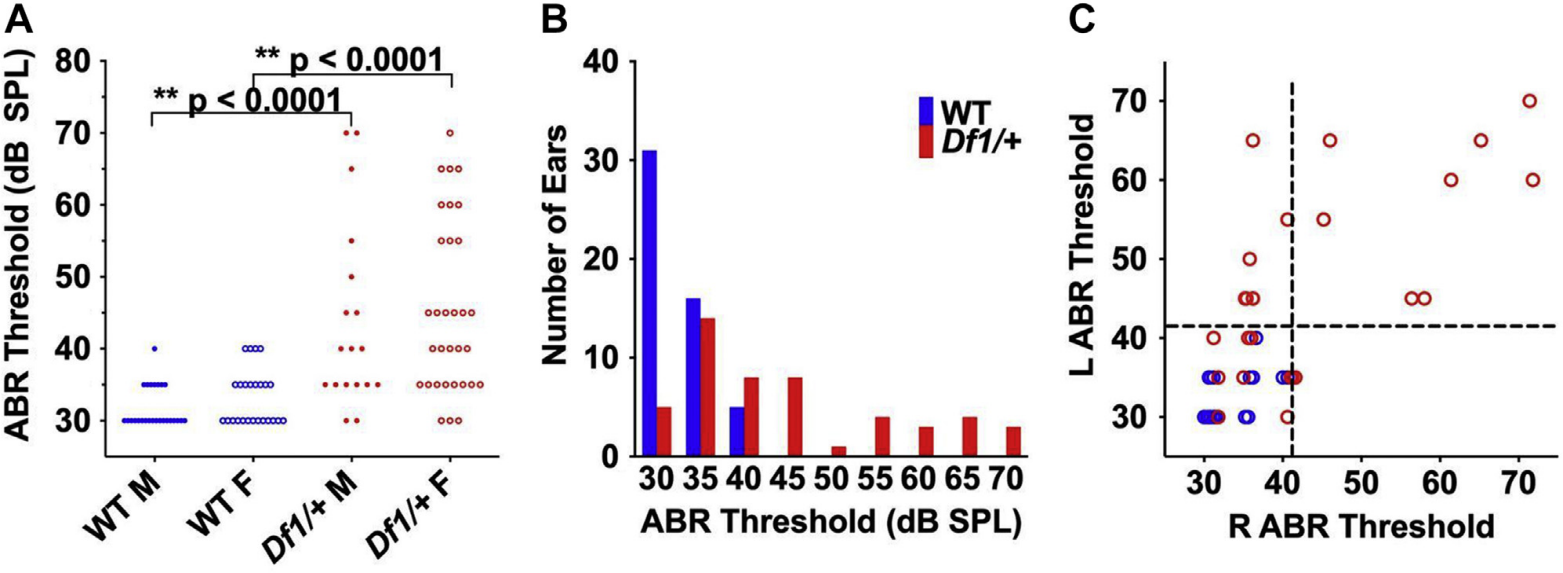
Elevated ABR thresholds in *Df1/+* mice. **(A)** Click ABR thresholds recorded from individual ears in male and female WT (blue) and *Df1/+* (red) mice. Data points represent individual ear measurements; animals typically contributed two measurements, one for each ear. *p* values indicate significant differences in Wilcoxon rank-sum tests (see text). Number of mice: 13 WT male, 13 WT female, 9 *Df1/+* male, 16 *Df1/+* female. **(B)** Click ABR thresholds pooled across recordings from male and female animals. Note that the *Df1/+* ABR threshold distribution extends from the minimum to well beyond the maximum of the WT range. **(C)** Relationship between left and right ear ABR thresholds in each mouse. Slight horizontal scatter added to aid visualization of overlapping data points. Dashed lines indicate the upper bound of normal hearing, defined as 2.5 standard deviations above the mean ABR threshold for WT ears. Upper right quadrant, binaural HI; upper left and lower right quadrant, monaural HI; lower left quadrant, normal hearing. Overall, 60% (15 of 25) of *Df1/+* mice had either monaural or binaural HI, and 46% (23 of 50) of *Df1/+* ears exhibited HI. Monaural HI in *Df1/+* mice occurred most commonly in the left ear, as observed previously (12). ABR, auditory brainstem response; F, female; HI, hearing impairment; L, left; M, male; R, right; SPL, sound pressure level; WT, wild-type.

**Figure 2:**
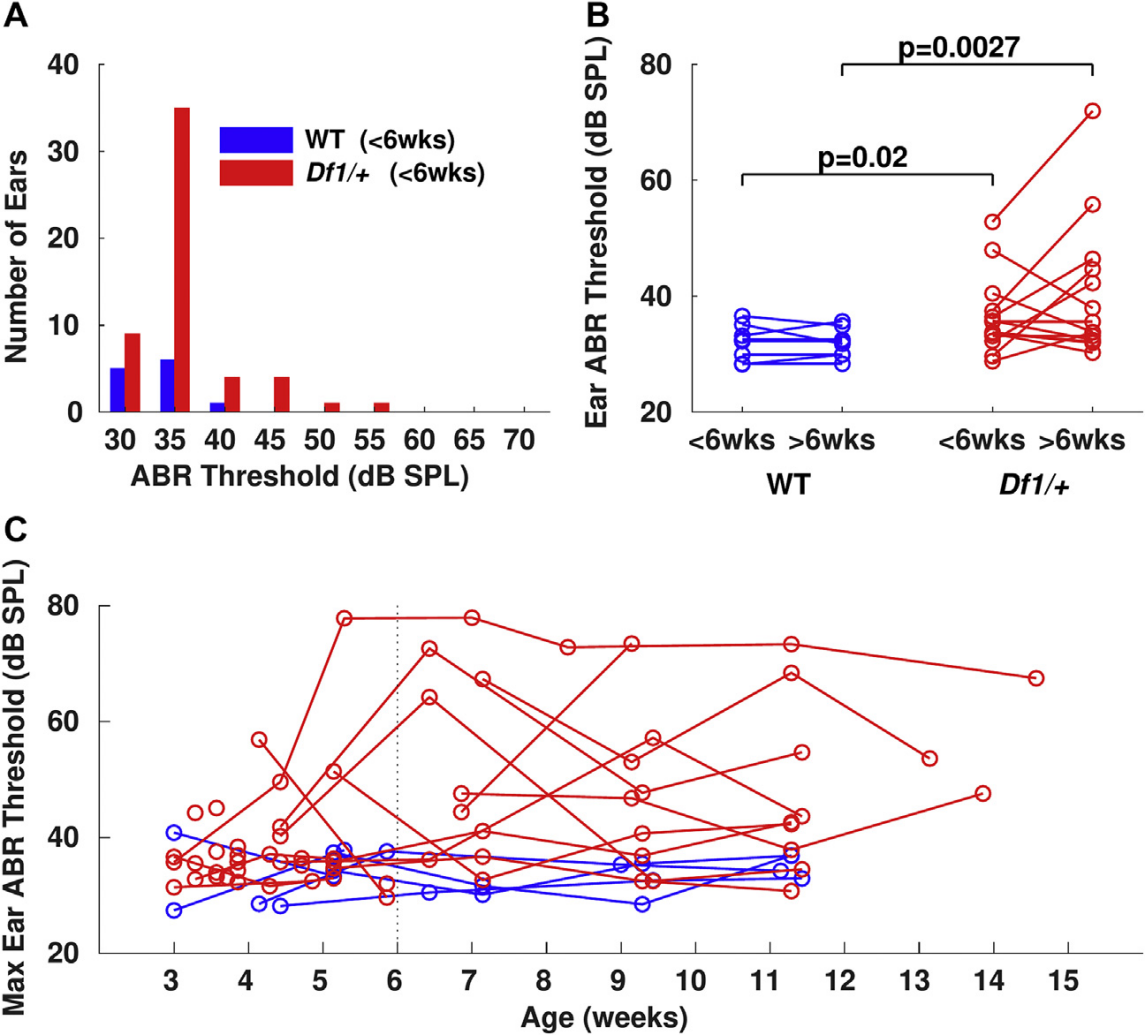
Hearing impairment typically emerges in a subset of *Df1/+* mice before 6 weeks of age and persists into adulthood. **(A)** Click ABR thresholds recorded in individual ears of WT (blue) and *Df1/+* (red) mice younger than 6 weeks old (i.e., before the typical age of sexual maturity). Data points represent individual ear ABR thresholds, averaged across any repeated measurements at ages younger than 6 weeks; each animal contributed two data points, one for each ear. Number of mice: 6 WT, 27 *Df1/+*. **(B)** Click ABR thresholds measured in the same mice at 3 to 6 weeks (<6 weeks) and 6 to 14 weeks (>6 weeks) of age. Individual ear threshold estimates were averaged across repeated measurements at different time points within either the <6 weeks age range (as in **[A]**) or the >6 weeks age range. Random vertical scatter (±3 dB SPL) added for display purposes only. *p* values indicate significant differences in Wilcoxon tests (see text). Number of mice: 4 WT, 7 *Df1/+*. **(C)** Maximum click-evoked ABR threshold across the two ears for each animal, shown for all measurement time points. Solid colored lines join repeated measurements from the same animal, where these could be obtained. Number of mice: 6 WT, 30 *Df1/+*. Random vertical scatter (±3 dB SPL) was added for display purposes only to help separate overlapping data points. ABR, auditory brainstem response; Max, maximum; SPL, sound pressure level; WT, wild-type.

All data collection and analysis was conducted blinded to animal genotype, except in experiments involving longitudinal ABR measurement, which involved mostly *Df1/+* mice. Separate measurements from the same animal obtained during auditory stimulation of left versus right ears were treated as independent measurements for some data analyses, because HI in *Df1/+* mice frequently affected only one ear. The distribution of ABR thresholds for *Df1/+* ears was bimodal rather than Gaussian (i.e., normal thresholds in some *Df1/+* ears, significant HI in others). Therefore, for comparisons involving ABR threshold measurements (Figure 1, Figure 2, and 7; Figure S3), we used nonparametric tests (Wilcoxon rank-sum or signed-rank tests for differences in medians of two unpaired or paired samples; Spearman’s rank correlation tests). Distributions of evoked-potential amplitudes and cell density were reasonably well approximated by Gaussian distributions. Therefore, for comparisons involving these measurements (Figure 4, Figure 5, Figure 6; Figures S1, S2, and S4), we used parametric tests (unpaired or paired *t* tests for differences in means of two unpaired or paired samples; ordinary one-way analysis of variance [ANOVA] for comparisons between multiple groups, followed by Fisher’s least significant difference [LSD] post hoc tests where group differences were significant). All statistical tests were two-tailed with α = 0.05.

**Figure 3:**
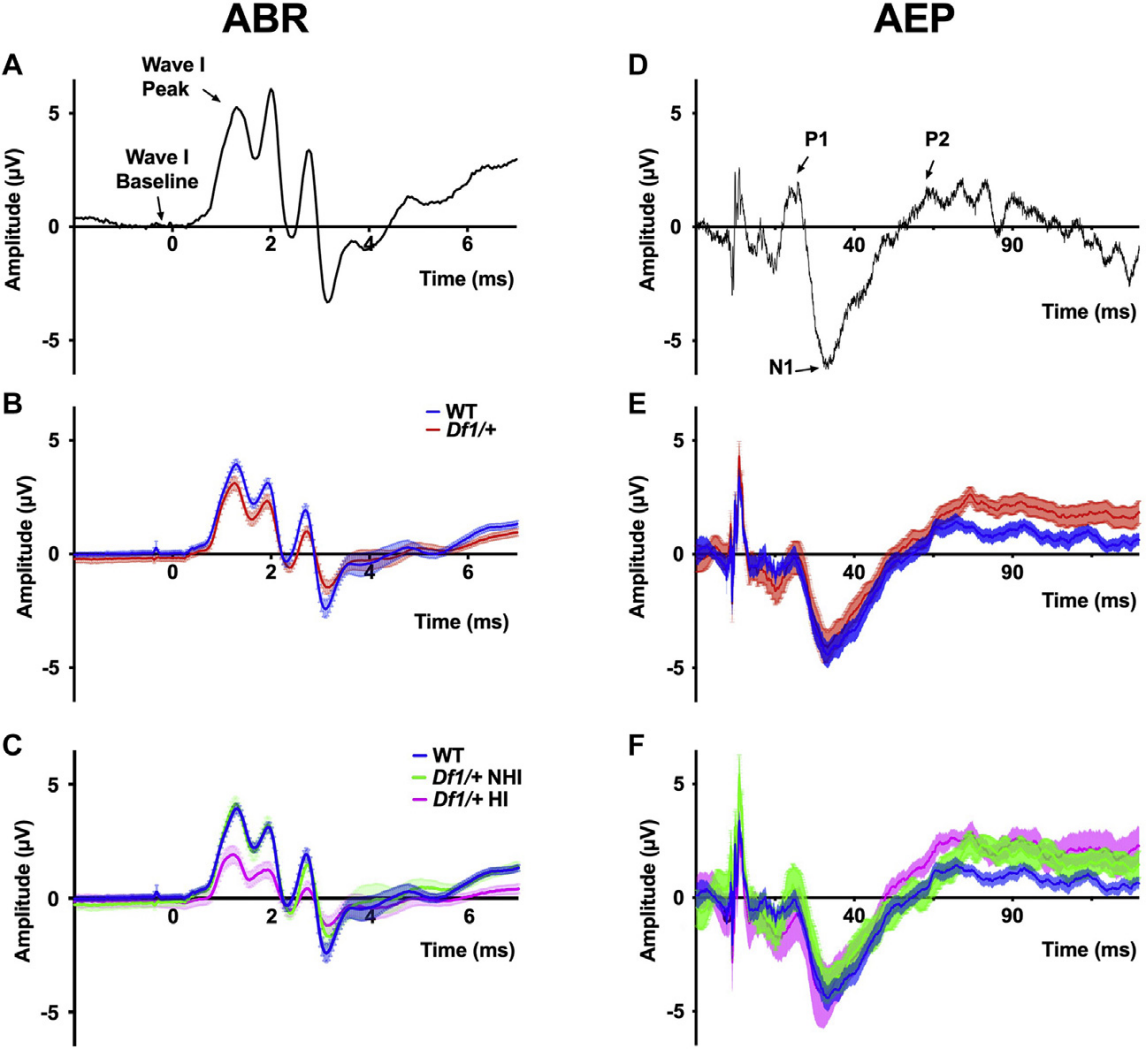
Average ABR and cortical AEP waveforms in WT and *Df1/+* mice. **(A)** Example trial-averaged ABR to an 80-dB SPL click, recorded ipsilateral to the stimulated ear in an individual WT animal. Arrows indicate baseline and peak used for measurement of wave I amplitude. **(B)** Mean ABR waveforms averaged across recordings from WT mice (blue) and *Df1/+* mice (red). Error bars indicate SEM across all trial-averaged ABR recordings for each group of animals. **(C)** Same as **(B)** but with *Df1/+* ABR recordings separated into those from *Df1/+* mice without HI (green, NHI) or *Df1/+* mice with HI in at least one ear (magenta, HI). **(D)** Example trial-averaged AEP to an 80-dB SPL click, recorded over auditory cortex contralateral to the stimulated ear in a WT animal. Arrows indicate P1 peak, N1 trough, and P2 peak. **(E)** Mean AEP waveforms averaged across recordings from different mice; color conventions as in **(B)**. Error bars indicate SEM across all trial-averaged AEP recordings for each group of animals. **(F)** Same as **(E)** but with *Df1/+* AEP recordings separated into those from *Df1/+* mice with or without HI in at least one ear; color conventions as in **(C)**. Ipsilateral ABR and contralateral AEP data were obtained from the same recording for each stimulated ear; however ABR waveforms in **(A–C)** represent differential signals while AEP waveforms in **(D–F)** are single-ended signals (see Methods and Materials). Each animal typically contributed two data points, one for each ear/hemisphere combination. Number of mice: 20 WT, 20 *Df1/+* (11 *Df1/+* NHI, 9 *Df1/+* HI). Number of ABR/AEP recordings: 39 WT, 40 *Df1/+* (22 *Df1/+* NHI, 18 *Df1/+* HI). ABR, auditory brainstem response; AEP, auditory evoked potential; HI, hearing impairment; NHI, no hearing impairment; SPL, sound pressure level; WT, wild-type.

**Figure 4:**
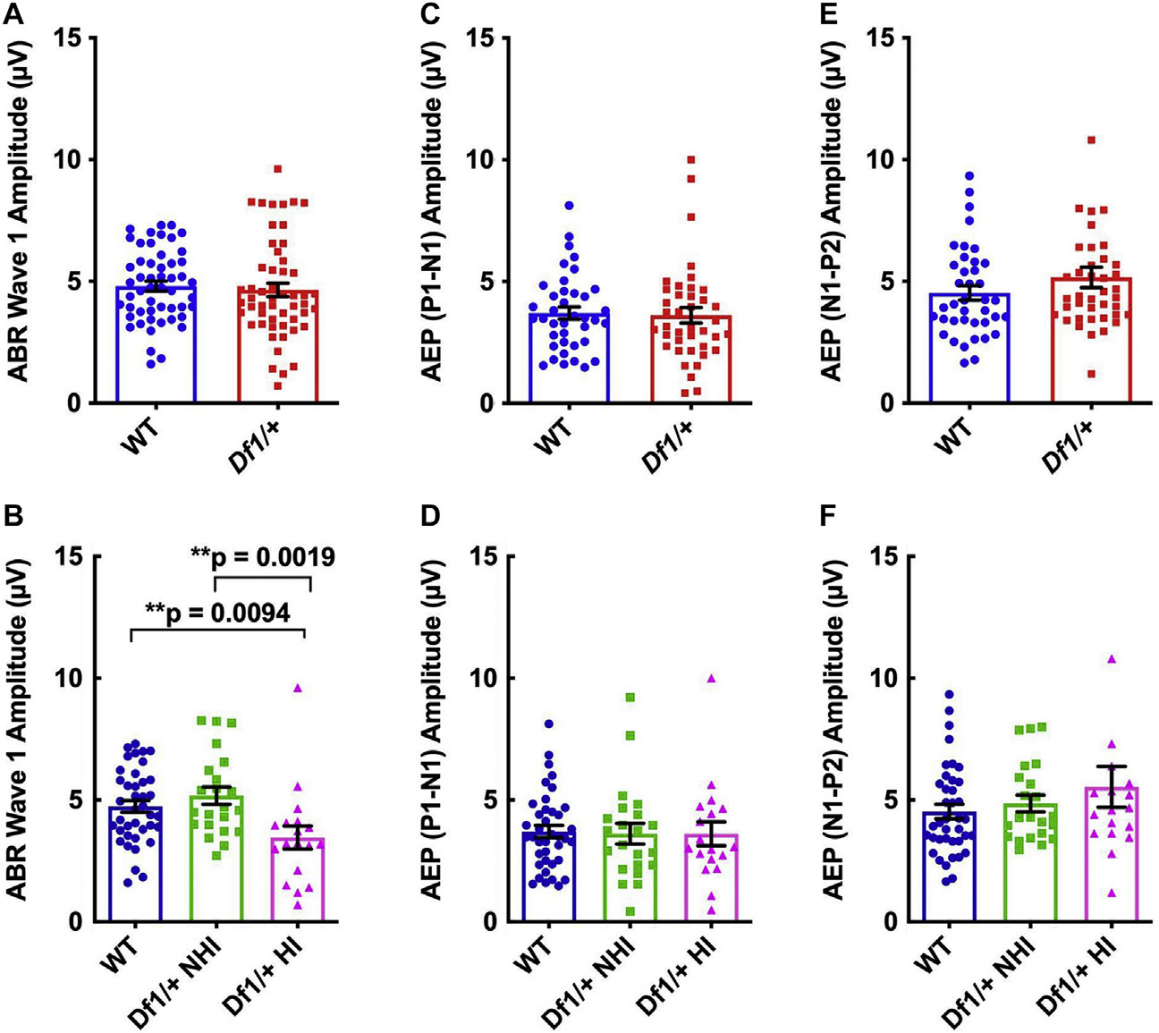
Reductions in ABR wave I amplitude in *Df1/+* mice with HI are not maintained in cortical AEPs. See text for details of statistical tests. **(A)** ABR wave I amplitude to an 80-dB SPL click does not differ between WT and *Df1/+* mice overall. **(B)** However, wave I amplitude to an 80-dB SPL click is reduced in *Df1/+* mice with HI relative to either WT mice or *Df1/+* mice without HI, while there is no significant difference in ABR wave I amplitude between *Df1/+* mice without HI and WT mice. **(C, D)** AEP P1-N1 amplitude does not differ between WT and *Df1/+* mice, either overall **(C)** or when *Df1/+* mice with and without HI are considered separately **(D)**. **(E, F)** No significant differences in AEP N1-P2 amplitude between WT and *Df1/+* mice, either overall **(E)** or when *Df1/+* mice with and without HI are considered separately **(F)**. Number of mice, number of ABR/AEP recordings, and color conventions as in Figure 3. Bars and error bars indicate mean ± SEM across recordings. ABR, auditory brainstem response; AEP, auditory evoked potential; HI, hearing impairment; NHI, no hearing impairment; SPL, sound pressure level; WT, wild-type.

**Figure 5:**
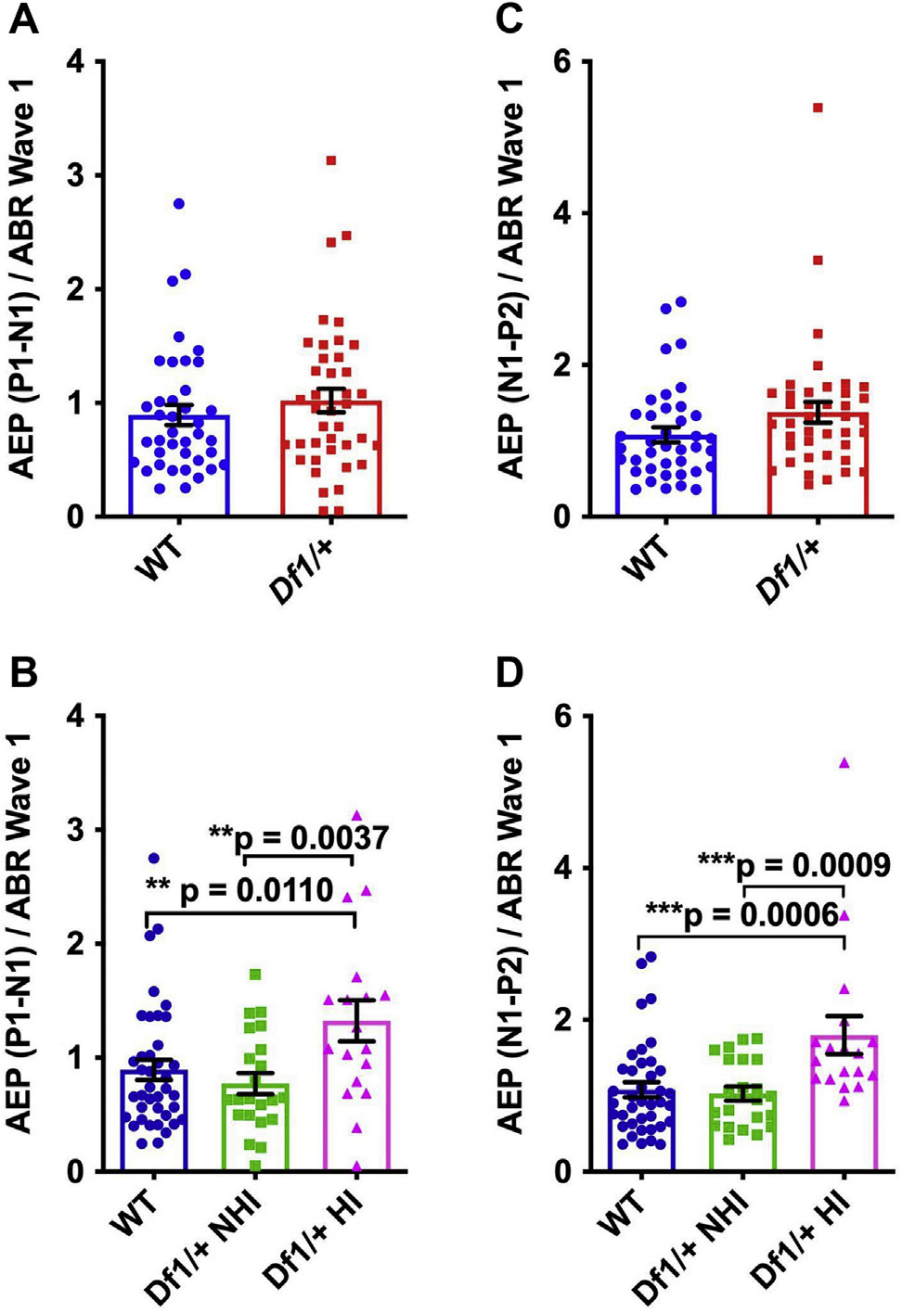
Central auditory gain is elevated in *Df1/+* mice with HI. See text for details of statistical tests. **(A)** The ratio of AEP P1-N1 amplitude to ABR wave I amplitude for an 80-dB SPL click does not differ between WT mice and *Df1/+* mice overall. **(B)** However, this measure of central auditory gain for the P1-N1 complex is significantly elevated in *Df1/+* mice with HI relative to either WT mice or *Df1/+* mice without HI. **(C)** Same as **(A)** but for the ratio of AEP N1-P2 amplitude to ABR wave I amplitude; no difference between WT mice and *Df1/+* mice overall. **(D)** Central auditory gain for the N1-P2 complex is significantly elevated in *Df1/+* mice with HI relative to either WT mice or *Df1/+* mice without HI. Number of mice and number of ABR/AEP recordings as in Figures 3 and 4; plot conventions as in Figure 4. ABR, auditory brainstem response; AEP, auditory evoked potential; HI, hearing impairment; NHI, no hearing impairment; SPL, sound pressure level; WT, wild-type.

**Figure 6:**
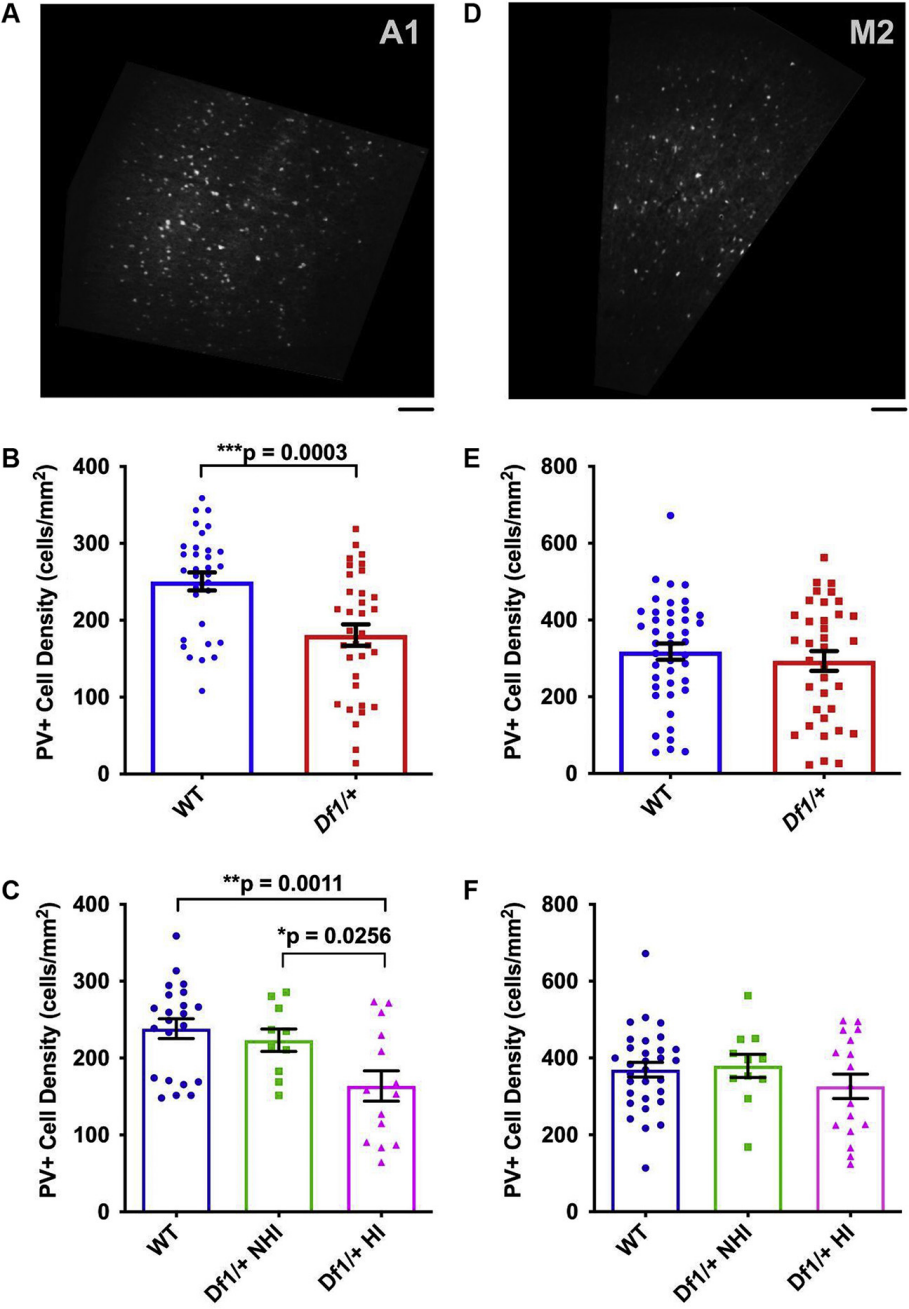
PV^+^ cell density is reduced in the auditory cortex but not the motor cortex of *Df1/+* mice. **(A)** Example confocal image used for cell counting. Coronal section through A1 stained with an antibody against the inhibitory interneuron marker PV. Areas outside A1 are masked in black. Scale bar: 0.1 mm. **(B)** PV^+^ cell density in A1 was significantly reduced in *Df1/+* mice overall compared with WT mice. (**C**) PV^+^ cell density in A1 was significantly reduced in *Df1/+* mice with HI compared with either WT mice or *Df1/+* mice without HI, but there was no significant difference between WT mice and *Df1/+* mice without HI. **(D)** Example PV-immunostained coronal section used for cell counting in M2. Areas outside M2 are masked in black. Scale bar as in **(A)**. **(E)** In M2, there was no significant difference in PV^+^ cell density between *Df1/+* and WT mice overall. **(F)** PV^+^ cell density in M2 also did not differ between groups when comparing WT mice, *Df1/+* mice without HI, and *Df1/+* mice with HI. See text for details of statistical tests and Table 1 for numbers of hemispheres (and mice) in each comparison. A1, primary auditory cortex; HI, hearing impairment; M2, secondary motor cortex; NHI, no hearing impairment; PV^+^, parvalbumin-positive; WT, wild-type.

**Figure 7:**
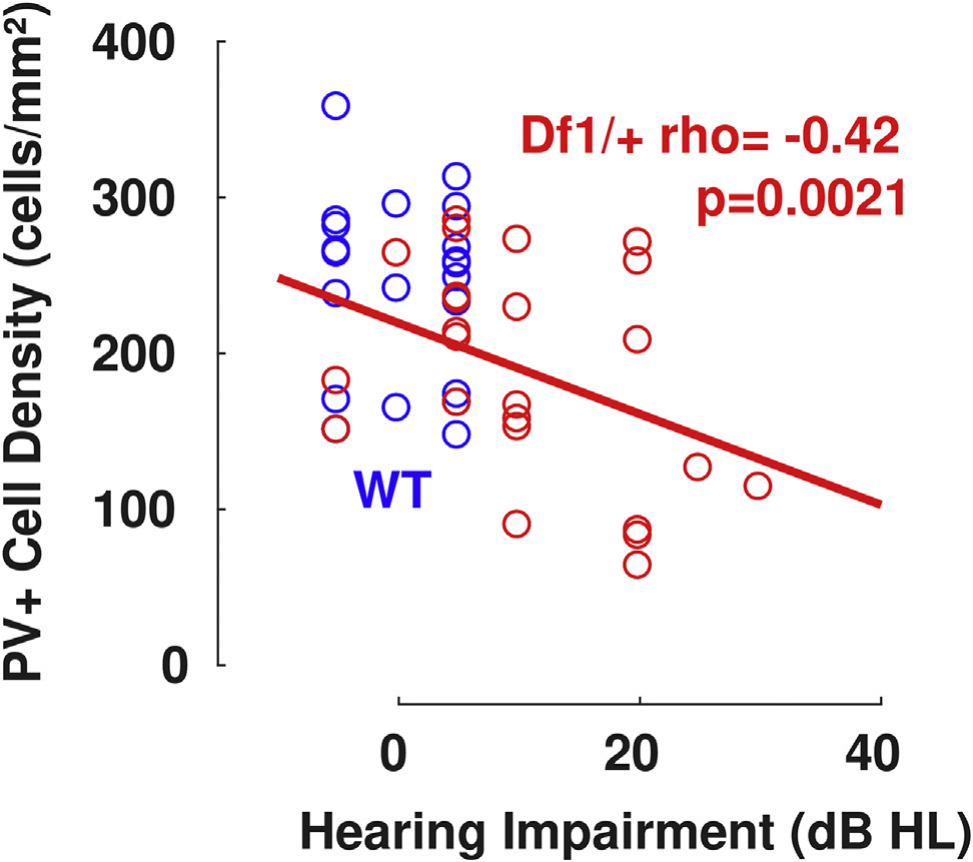
PV^+^ cell density in the auditory cortex correlates inversely with hearing impairment in *Df1/+* mice. Each data point (blue, WT; red, *Df1/+*) represents a PV^+^ cell density measurement from A1 in the left or right hemisphere; individual mice typically contributed two measurements, one for each hemisphere. Here, hemisphere measurements are plotted against an overall measure of hearing impairment for each animal (x-axis), obtained by calculating the maximum click-evoked auditory brainstem response threshold across ears and then subtracting the mean of these values across WT animals only. For comparisons of PV^+^ cell density in each hemisphere to left, right, contralateral, and ipsilateral ear auditory brainstem response thresholds, see Figure S3. Red text, Spearman’s rho and *p* value for correlation of PV^+^ cell density with hearing impairment for *Df1/+* mice only. Solid red line, two-dimensional least squares linear fits to the *Df1/+* data. Number of mice and number of hemispheres as in Table 1 and Figure 6. dB HL, decibels hearing loss relative to normal hearing threshold; PV^+^, parvalbumin-positive; WT, wild-type.

## Results

### Approximately 60% of Adult *Df1/+* Mice Have HI in One or Both Ears

We quantified hearing sensitivity in adult *Df1/+* and WT mice aged 6.6 to 24.6 weeks (overall median = 10.1 weeks) using the click-evoked ABR as in previous work (12). There was no significant difference in age between the two groups (median [95% CI]: *Df1/+*, 8.3 [6.6–19.3] weeks; WT, 10.4 [6.6–24.6] weeks; Wilcoxon rank-sum test, *p* = .06). ABR thresholds were obtained for each ear in each mouse, except in one WT animal that died after measurement in only one ear.

Replicating previous results obtained in a cohort of older *Df1*/+ and WT mice (12), we found clear evidence for HI in more than half of the *Df1*/+ animals (Figure 1). Median ABR thresholds were significantly higher in *Df1/+* than WT mice (median [95% CI]: *Df1/+*, 40 [30–70] dB SPL; WT, 30 [30–40] dB SPL; Wilcoxon rank-sum test, *p* < .0001). Elevation of ABR thresholds in *Df1*/+ relative to WT mice was evident in both male and female animals, but there were no differences between genders within genotype (Wilcoxon rank-sum tests, *p* < .0001 between genotypes within gender, *p* > .1 between genders within genotype) (Figure 1A).

There was substantial between-ear and interindividual variation in hearing ability among *Df1/+* animals (Figure 1B), i.e., ABR thresholds were abnormally elevated in some *Df1*/+ ears but not others. Defining the upper bound of normal hearing as 2.5 SD above the mean ABR threshold for WT ears (i.e., abnormal hearing threshold: >40.88 dB SPL), we found that 46% (23 of 50) of *Df1*/+ ears and 0% (0 of 51) of WT ears displayed HI. Overall, 60% (15 of 25) of *Df1*/+ mice had either monaural or binaural HI, and monaural HI occurred most commonly in the left ear (Figure 1C). These results align both qualitatively and quantitatively with findings previously reported in *Df1/+* and WT animals tested at similar or older ages (12).

### HI Emerges Before Adulthood in *Df1/+* Mice

To investigate the early timecourse of HI in *Df1/+* mice, we measured click and tone ABR thresholds in mice as young as 3 weeks of age (i.e., at weaning) and conducted a longitudinal study of changes in ABR thresholds over time. This work was performed using a separate cohort of *Df1/+* and WT mice bred and tested in a different facility and country (at the National Institutes of Health, United States, instead of University College London, United Kingdom).

HI was evident in a subset of *Df1/+* mice well before adulthood. Even among mice <6 weeks old, i.e., before puberty in mice (28), click ABR thresholds were elevated in *Df1/+* ears compared with WT ears (median [95% CI]: *Df1/+* 35 [30–45] dB SPL vs. WT 32.5 [30–37.2] dB SPL; Wilcoxon rank-sum test, *p* = .012) (Figure 2A). Moreover, early HI tended to persist in affected ears. In mice for which click ABR measurements could be obtained at both young and adult ages (<6 weeks and >6 weeks), we found significant differences in ear ABR thresholds between *Df1/+* and WT animals within both age groups, but no significant differences between ages within genotype (Wilcoxon rank-sum tests, *Df1/+* vs. WT: <6 weeks, *p* = .020; >6 weeks, *p* = .0027; Wilcoxon signed-rank tests, <6 vs. >6 weeks: *Df1/+*, *p* = .26; WT, *p* = 1) (Figure 2B).

Thus, HI emerged early in a subset of *Df1/+* animals and typically persisted for many weeks once it emerged. Longitudinal measurements of maximum ear click ABR thresholds for individual mice revealed multiple examples of *Df1/+* mice with early-onset and persistent HI, along with examples of *Df1/+* mice with normal hearing across all tested ages (Figure 2C). Similar results were also obtained when measuring ABR thresholds using 8 or 16 kHz tones.

### ABR Wave I Amplitude Reductions in *Df1*/+ Mice With HI Are Not Maintained in Cortical AEPs

We wondered if auditory brain responses might differ between *Df1*/+ and WT mice and whether any differences might be related to the HI afflicting a subset of *Df1/+* animals. Previous studies have reported abnormalities in sound-evoked auditory thalamic and/or cortical activity in mouse models of 22q11.2DS, but have either not investigated the role of HI (11) or not observed HI in the animals tested (29).

We recorded both ABR waves and contralateral cortical AEP waves following presentations of loud suprathreshold (80 dB SPL) clicks at 300-ms interclick intervals in adult mice from the Figure 1 cohort. To assess the timing and strength of afferent input to the auditory brain, we measured the peak latency and baseline-to-peak amplitude of ABR wave I (Figure 3A), which arises from the auditory nerve. Within the AEP, we focused on wave peaks or troughs typically attributed to activity within the auditory thalamus (P1), auditory cortex (N1), and associative cortices (P2), measuring P1, N1, and P2 latency and P1-N1 and N1-P2 amplitudes (Figure 3D).

HI, defined here as an elevation of the ABR threshold, would be expected to reduce ABR wave I amplitude for a suprathreshold click. Indeed, the amplitude of ABR wave I to an 80-dB SPL click was significantly lower in *Df1*/+ mice with HI than in either *Df1*/+ mice with no HI (NHI) or WT mice (one-way ANOVA, *F*_2,76_ = 5.55, group difference *p* = .0056; Fisher’s LSD, *Df1/+* HI vs. WT *p* = .0094, *Df1/+* HI vs. *Df1/+* NHI *p* = .0019) (Figures 3C and 4B). However, there was no significant difference in ABR wave I amplitude between *Df1*/+ NHI mice and WT animals (unpaired *t* test, *p* = .33) (Figures 3C and 4B) nor between *Df1*/+ and WT mice overall (unpaired *t* test, *p* = .66) (Figures 3B and 4A).

More surprisingly, there were no significant differences between *Df1*/+ and WT mice in either P1-N1 or N1-P2 cortical AEP wave amplitudes, even when *Df1*/+ mice with and without HI were considered separately (unpaired *t* test, WT vs. *Df1/+* overall, P1-N1: *p* = .82 and N1-P2: *p* = .22; one-way ANOVA, WT vs. *Df1/+* NHI vs. *Df1/+* HI, group differences P1-N1: *F*_2,76_ = 0.025, *p* = .98 and N1-P2: *F*_2,76_ = 1.20, *p* = .31) (Figures 3E, F and 4C–F). There were also no significant differences in latencies of ABR wave I or cortical AEP waves P1, N1, or P2 between WT and *Df1*/+ animals, either overall or when HI in *Df1*/+ mice was taken into account (Figure S1).

Thus, while ABR wave I amplitude was reduced as expected in *Df1*/+ mice with HI, there were no significant differences in the cortical AEP waves between any of the subgroups. This result suggests an increase in central auditory gain in *Df1*/+ mice with HI, as previously observed in animal models of more profound, bilateral hearing loss [e.g., (16,30)].

### Central Auditory Gain Is Elevated Specifically in *Df1*/+ Mice With HI

To quantify central auditory gain, we compared ABR wave I amplitude to cortical AEP P1-N1 or N1-P2 amplitude recorded simultaneously over the contralateral cortical hemisphere. We used the ratios of cortical AEP P1-N1 or N1-P2 amplitude to ABR wave I amplitude as measures of central auditory gain.

Both the P1-N1 and N1-P2 gain measures revealed elevated central auditory gain specifically in *Df1*/+ mice with HI (Figure 5). When comparing *Df1/+* mice overall with WT mice, we observed no significant differences in the ratio of either AEP P1-N1 amplitude or N1-P2 amplitude to ABR wave I amplitude (unpaired *t* test, P1-N1: *p* = .36 and N1-P2: *p* = .084) (Figure 5A, C). However, the P1-N1 gain measure was significantly higher in *Df1*/+ mice with HI than in either WT mice or *Df1*/+ NHI mice, while *Df1*/+ NHI mice were not significantly different from WT animals (one-way ANOVA, *F*_2,76_ = 4.96, group difference *p* = .0094; Fisher’s LSD, *Df1/+* HI vs. WT *p* = .011, *Df1/+* HI vs. *Df1/+* NHI *p* = .0037, WT vs. *Df1/+* NHI *p* = .44) (Figure 5B). Similar results were obtained for the N1-P2 gain measure (one-way ANOVA, *F*_2,76_ = 7.68, group difference *p* = .0009; Fisher’s LSD, *Df1/+* HI vs. WT *p* = .0006, *Df1/+* HI vs. *Df1/+* NHI *p* = .0009, WT vs. *Df1/+* NHI *p* = .79) (Figure 5D). These results suggest that central auditory abnormalities arise in some *Df1/+* mice as a consequence of HI.

### *Df1*/+ Mice With HI Have Reduced Density of PV^+^ Interneurons in the Auditory Cortex

Changes in central auditory gain following HI have been linked with alterations in PV^+^ interneuron activity in the auditory cortex (31,32), and abnormalities in PV^+^ interneuron networks are also a common finding in animal models of schizophrenia [see (33,34) for reviews]. We wondered whether auditory cortical PV^+^ interneuron density might be abnormal in *Df1*/+ mice and, if so, how these abnormalities might relate to HI. To examine both PV^+^ cell density and density of neurons overall, we performed immunohistochemical staining for PV and NeuN (a pan-neuronal marker) in coronal brain sections through the auditory cortex (Figure 6A; Figure S2A) in adult *Df1/+* and WT mice, most of which had also undergone ABR testing (Table 1).

PV^+^ cell density in the auditory cortex was significantly lower in *Df1/+* than WT mice (unpaired *t* test, *p* = .00030) (Figure 6B), and this difference arose primarily from abnormalities in the subset of *Df1/+* mice with HI. In *Df1*/+ mice with HI, PV^+^ cell density was significantly lower than in either WT mice or *Df1*/+ NHI mice, while PV^+^ cell density in *Df1*/+ NHI mice did not differ from that in WT animals (one-way ANOVA, *F*_2,43_ = 6.37, group difference *p* = .0038; Fisher’s LSD, *Df1/+* HI vs. WT *p* = .0011, *Df1/+* HI vs. *Df1/+* NHI *p* = .026, WT vs. *Df1/+* NHI *p* = .53) (Figure 6C). In contrast, there were no significant differences in NeuN^+^ cell density in A1 between WT and *Df1*/+ animals with or without HI (Figure S2B, C).

PV^+^ interneuron density in the auditory cortex was inversely correlated with the severity of HI in *Df1/+* mice (Figure 7). We quantified the degree of HI in each mouse by calculating the maximum click-evoked ABR threshold across ears and then subtracting the average of these values across WT animals. In *Df1/+* mice, auditory cortical PV^+^ cell density decreased as the degree of HI increased (Spearman’s ρ = −0.42, *p* = .0021) (Figure 7). Similar trends were evident when PV^+^ cell density in auditory cortical hemispheres from *Df1/+* mice was compared with left, right, contralateral, or ipsilateral ear ABR thresholds, with a significant negative correlation for the relationship with left ear ABR threshold in particular (Figure S3). Thus, PV^+^ interneuron abnormalities in the auditory cortex of *Df1/+* mice are related to the variable HI observed in these animals.

### *Df1*/+ Mice Do Not Show Abnormalities in PV^+^ or NeuN^+^ Cell Density in M2 or Changes in Laminar Distribution of PV^+^ Cells in A1

The auditory cortex in mice is reciprocally connected with the secondary motor area (M2) in the frontal cortex, and neural activity in M2 is known to modulate auditory cortical processing (35,36). To find out if reductions in PV^+^ cell density observed in A1 of *Df1*/+ mice with HI also occurred in M2, we analyzed PV and NeuN immunostaining in coronal brain sections through the frontal cortex (Figure 6D; Figure S2D).

Results suggest that reductions in PV^+^ cell density in *Df1/+* mice with HI may be specific to the auditory cortex. PV^+^ cell density in M2 did not differ between WT and *Df1*/+ mice (unpaired *t* test, *p* = .46) (Figure 6E) or between WT mice, *Df1*/+ NHI mice, and *Df1*/+ mice with HI (one-way ANOVA, *F*_2,56_ = 1.04, group difference *p* = .36) (Figure 6F). There were also no significant differences between animal groups in NeuN^+^ cell density in M2 (Figure S2E, F).

Furthermore, we observed no abnormalities in laminar distribution of PV^+^ interneurons in A1 of *Df1/+* mice and minimal evidence for abnormalities in M2. Comparing WT mice with *Df1/+* mice overall, we found no significant differences in the cortical depth distribution of PV^+^ interneurons in either A1 or M2 (Figure S4A, C). Comparing WT mice and *Df1*/+ mice with and without HI, we again found no significant differences in depth distribution of PV^+^ cells in A1, while a weak effect of HI was observed in M2 (Figure S4B, D). Post hoc tests identified the significant result in M2 as arising from a reduction in PV^+^ cell density in *Df1/+* mice with HI at cortical depths 0.6 to 0.8 of the total distance from pia-to-white matter. This slight alteration in M2 PV^+^ cell distribution in *Df1/+* mice with HI is reminiscent of aberrant laminar distributions of PV^+^ cells previously observed in medial cortical regions of the *LgDel* mouse (37,38). However, in M2 of *Df1/+* mice, the laminar abnormalities appeared relatively weak, despite a comparatively large sample size.

## Discussion

Our results show that abnormalities in cortical AEPs and PV^+^ interneuron density in the *Df1/+* mouse model of 22q11.2DS are related to the degree of peripheral HI in individual *Df1/+* animals. In principle, this correlation could arise from either direction of a causal relationship between auditory brain abnormalities and peripheral HI, or from a common underlying cause that varies across *Df1/+* animals despite their genetic similarity.

The most plausible of the two possible causal relationships is that auditory brain abnormalities in *Df1/+* mice are caused either entirely by peripheral HI or by an interaction between HI and other genetic vulnerabilities associated with the 22q11.2 deletion. Peripheral HI in *Df1/+* mice arises from middle-ear inflammation (otitis media) triggered by developmental defects in muscles of the Eustachian tube (12,13). It is highly unlikely that auditory cortical abnormalities in *Df1/+* mice alter peripheral hearing sensitivity; even bilateral auditory cortex lesions do not affect ABR thresholds in mice (38). In contrast, experimentally induced peripheral HI is already known to increase central auditory gain and to alter cortical excitation/inhibition balance in mice and other animals (18,31,39, 40, 41).

It is also possible that individual differences in auditory brain abnormalities and peripheral HI among *Df1/+* mice arise from a common underlying cause, such as varying levels of inflammation. Distinguishing the common-cause explanation from causal effects of HI will require further experiments in other mouse models of otitis media and in WT mice with induced HI.

### HI in Mouse Models of 22q11.2DS

Our data confirm that approximately 60% of adult *Df1*/+ mice have HI in one or both ears (12) and demonstrate for the first time that HI emerges well before adulthood in affected animals. These observations raise the possibility of developmental as well as acute effects of HI on brain function in a subset of *Df1/+* mice. Even HI that occurs only in one ear can drive plastic changes throughout the central auditory system, particularly if it occurs during development (17,20,21,42,43).

HI in *Df1/+* mice has previously been shown to correlate ear-by-ear with otitis media (12), which is also the primary cause of HI in humans with 22q11.2DS (5). In *Df1/+* mice, susceptibility to otitis media arises from a developmental defect in a muscle affecting drainage of the middle ear through the Eustachian tube (13). This muscle defect is caused by haploinsufficiency of the gene *Tbx1* (13,44); in humans, *TBX1* lies within the minimum 22q11.2 deletion region. Thus, our results and previous work (12,13) suggest that any mouse model of 22q11.2DS with heterozygous deletion of *Tbx1* may be susceptible to otitis media and HI from an early age.

There are, however, some discrepant results in the literature; two previous studies that tested peripheral hearing sensitivity in mouse models of 22q11.2DS found no significant differences from WT animals (29,45). Age differences or genetic differences in the mice seem unlikely to explain the discrepant results, because HI is evident even in young *Df1/+* mice and arises from *Tbx1* haploinsufficiency. Differences in the microbiological status of the mice seem a more plausible explanation, given that opportunistic pathogens in laboratory mouse facilities can increase risk of otitis media (46). Mice used in this study were bred and maintained in standard mouse housing facilities. It is possible that the incidence of otitis media and HI in *Df1*/+ mice might be lower in superclean facilities. However, HI and otitis media have been found to affect a majority of human patients with 22q11.2DS (5). Therefore, even if it were possible to reduce the incidence of otitis media in *Df1*/+ mice by restricting their microbiological exposure, the resulting animals would be poorer models of the human syndrome (47).

### Central Auditory Abnormalities in Mouse Models of 22q11.2DS

We found that measures of central auditory gain (e.g., ratios between AEP P1-N1 or N1-P2 amplitude and ABR wave I amplitude) were significantly higher in *Df1*/+ mice with HI than in WT mice or in *Df1*/+ NHI mice. This finding is consistent with previous literature on effects of HI. Loss of peripheral auditory input drives homeostatic changes throughout the auditory brainstem, midbrain, thalamus, and cortex, which typically manifest as reductions in inhibitory synaptic transmission, increased spontaneous activity, and increased gain of sound-evoked responses (16,18,21,31,48, 49, 50, 51). Thus, increased central auditory gain in *Df1/+* mice with HI could arise at multiple stages of the central auditory pathway.

### PV^+^ Cortical Interneurons and HI

We observed a reduction in the density of PV-expressing cortical interneurons in *Df1/+* mice, which was specific to the auditory cortex and correlated with degree of HI in individual animals.

To our knowledge, this is not only the first report of a link between HI and reduced PV^+^ interneuron density in an animal model of schizophrenia but also the first indication that HI due to otitis media may influence PV^+^ interneuron density in the auditory cortex. Previous studies in mice have demonstrated that PV^+^ interneuron density and distribution in the auditory cortex can be affected by age-related changes in the auditory system (52, 53, 54, 55), by noise-induced or pharmacologically induced sensorineural hearing loss (32,54), and by mutations that disrupt both auditory hair cell function and cortical interneuron migration (56). Our findings raise the additional possibility that conductive HI, either alone or in combination with genetic risk factors for schizophrenia, may lead to reductions in PV^+^ interneuron density.

Reduced PV^+^ cell density could arise from disrupted PV^+^ cell migration to cortex during embryonic development (embryonic days 13–17), increased PV^+^ cell death during postnatal development (postnatal days 5–15), and/or reduction in PV expression in cortical interneurons after development (57,58). Previous work suggests that otitis media develops soon after ear opening (at postnatal day 11) in affected *Df1/+* ears (13), a finding consistent with our observation of HI in *Df1/+* mice at 3 to 6 weeks of age. Therefore, HI in *Df1/+* mice likely emerges after embryonic migration of PV^+^ cells but might affect postnatal PV^+^ cell development and PV expression in adulthood. Further experiments are required to determine the timing of the reduction in PV^+^ cell density in the auditory cortex.

### PV^+^ Cortical Interneurons in Mouse Models of 22q11.2DS

PV^+^ interneurons are known to play a pivotal role in maintenance of normal cortical circuit function (59, 60, 61). Previous studies of mouse models of 22q11.2DS have reported altered laminar distribution and/or reduced density of PV^+^ interneurons in the medial prefrontal cortex and hippocampus (37,57,58,62,63). Abnormalities in PV^+^ interneurons are also a common finding in human schizophrenia and are thought to contribute to cognitive deficits (25,64, 65, 66, 67). Thus, auditory cortical PV^+^ interneuron abnormalities in mouse models of 22q11.2DS seem likely to be relevant to understanding cortical circuit dysfunction in schizophrenia and the influence of other potential risk factors, such as HI.

### Implications for Schizophrenia Research

In humans, there is compelling evidence that HI increases the risk of psychosis and hallucinations [see (15) for a recent meta-analysis and review]. Moreover, HI and/or middle-ear disease in childhood is associated with elevated risk of developing schizophrenia in adulthood (15,68, 69, 70). The mechanisms underlying this association are unknown but could include changes in neuronal networks driven by loss of sensory input. In individuals with genetic vulnerability to schizophrenia, including but not only patients with 22q11.2DS, HI from middle-ear problems might be a critical second hit that breaks the balance of excitation and inhibition in the cortex and promotes development of hallucinations and other schizophrenia symptoms. Our results demonstrate that the *Df1/+* mouse model of 22q11.2DS is an ideal system for studying how genetic vulnerability to schizophrenia, HI, and/or interactions between these factors could produce brain abnormalities that promote psychiatric disease.

## Supplementary Material

Supplementary Material

Key Resources Table
