# Supplementary material for: Increased Central Auditory Gain and Decreased Parvalbumin-Positive Cortical Interneuron Density in the Df1/+ Mouse Model of Schizophrenia Correlate With Hearing Impairment
Source: Biol Psychiatry Glob Open Sci. 2022 Mar 16;3(3):386–97. doi: 10.1016/j.bpsgos.2022.03.007 (PMC10382707; doi:10.1016/j.bpsgos.2022.03.007)
Supplement: Supplementary Material [file mmc1.pdf]

SUPPLEMENTARY INFORMATION

Increased Central Auditory Gain and Decreased Parvalbumin-Positive Cortical Interneuron Density in the *Df1/+* Mouse Model of Schizophrenia Correlate With Hearing Impairment

SUPPLEMENTARY FIGURES

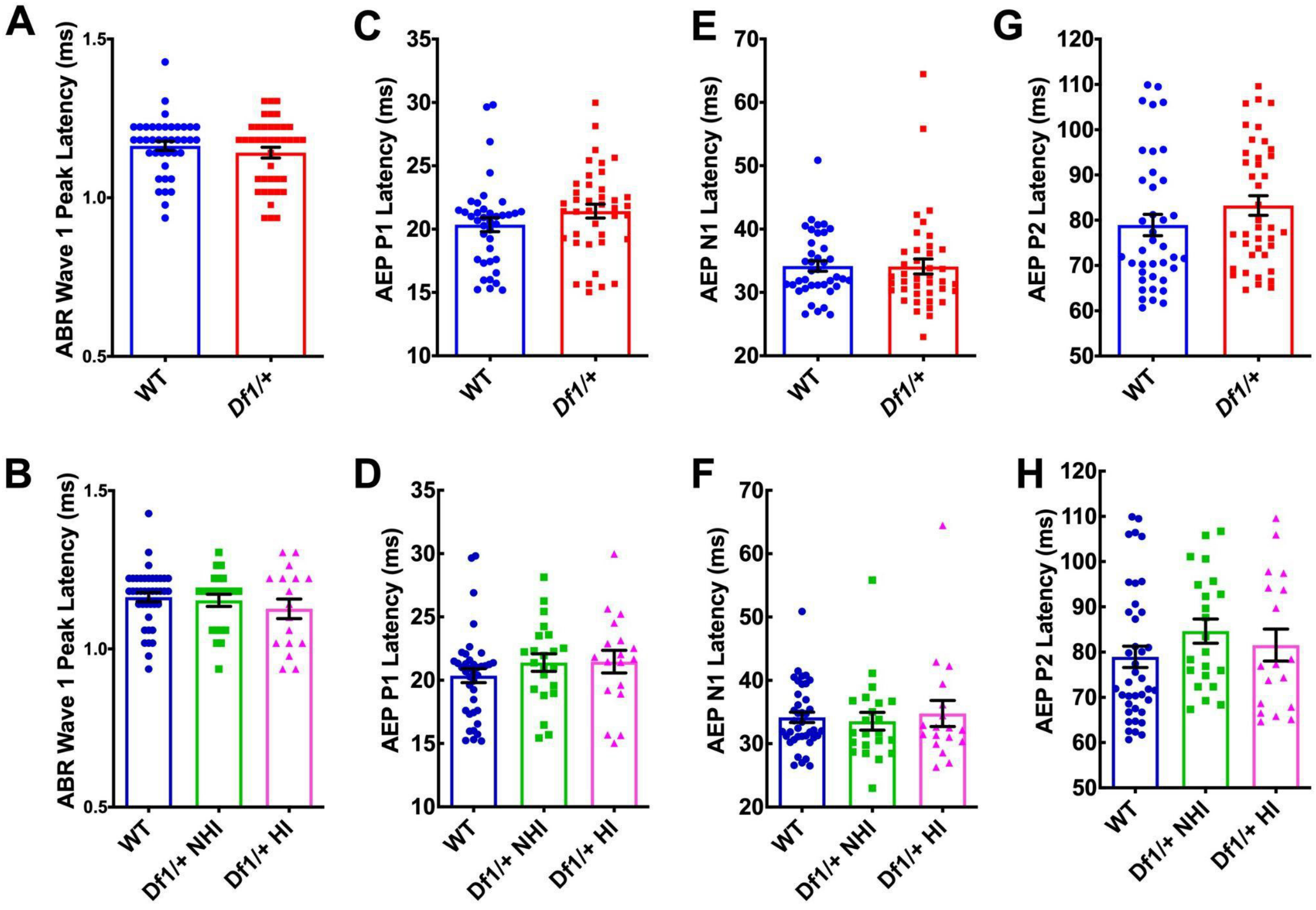

Figure S1. No significant differences in ABR or cortical AEP wave latencies between *Df1/*+ and WT mice.

(A-B) Latency of ABR wave I peak. No significant differences between WT and *Df1/*+ mice overall (A; unpaired *t*-test, p=0.34), nor between WT mice, *Df1/*+ mice without hearing impairment, and *Df1/*+ mice with hearing impairment (B; one-way ANOVA, F(2,76)=0.81, group difference p=0.45).

(C-D) Latency of cortical AEP wave P1 peak. No significant differences between WT and *Df1/*+ mice overall (C; unpaired *t*-test, p=0.17), nor between WT mice, *Df1/*+ mice without hearing impairment, and *Df1/*+ mice with hearing impairment (D; one-way ANOVA, F(2,76)=0.94, group difference p=0.39).

(E-F) Latency of cortical AEP wave N1 peak. No significant differences between WT and *Df1/*+ mice overall (E; unpaired *t*-test, p=0.97), nor between WT mice, *Df1/*+ mice without hearing impairment, and *Df1/*+ mice with hearing impairment (F; one-way ANOVA, F(2,76)=0.18, group difference p=0.84).
(G-H) Latency of cortical AEP wave P2 peak. No significant differences between WT and *Df1/*+ mice overall (G; unpaired *t*-test, p=0.18), nor between WT mice, *Df1/*+ mice without hearing impairment, and *Df1/*+ mice with hearing impairment (F; one-way ANOVA, F(2,76)=1.14, group difference p=0.33).
Number of mice and number of ABR/AEP recordings as in Figures 2 and 3; plot conventions as in Figure 3.

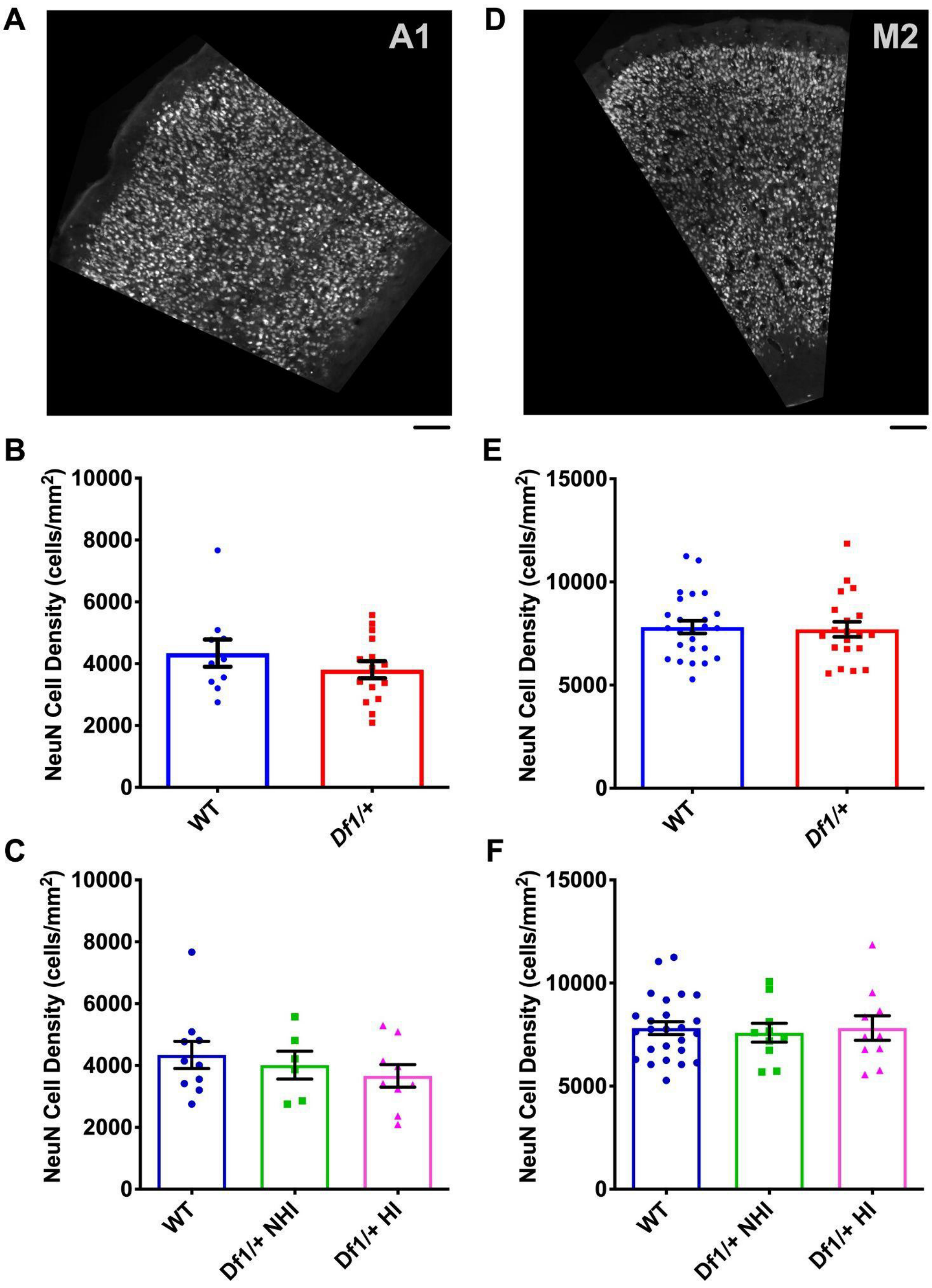

Figure S2. No significant differences in NeuN+ cell density between WT and *Df1/+* mice, in either the auditory cortex or the motor cortex.

(A) Example confocal image of a coronal section through primary auditory cortex (A1) stained with an antibody against the pan-neuronal marker NeuN. Areas outside A1 are masked in black. Cell counting was

performed within a pia-to-white-matter rectangular strip through the center of A1 (5% of total area). Scale bar: 0.1 mm.

(B) NeuN+ cell density in A1 did not differ between WT and *Df1/+* mice overall (unpaired t-test, p=0.29).

(C) NeuN+ cell density in A1 also did not differ between groups when comparing WT mice, *Df1/+* mice without hearing impairment, and *Df1/+* mice with hearing impairment (one-way ANOVA, F(2,22)=0.72, group difference p=0.50).

(D) Example NeuN-immunostained coronal section through secondary motor cortex (M2). Areas outside M2 are masked in black. Cell counting was performed within a pia-to-white-matter rectangular strip through the center of M2 (5% of total area). Scale bar as in A.

(E) NeuN+ cell density in M2 did not differ between WT and *Df1/+* mice overall (unpaired t-test, p=0.82).

(F) NeuN+ cell density in M2 also did not differ between groups when comparing WT mice, *Df1/+* mice without hearing impairment, and *Df1/+* mice with hearing impairment (one-way ANOVA, F(2,42)=0.076, group difference p=0.93).

See Table 1 for numbers of hemispheres and mice in each comparison.

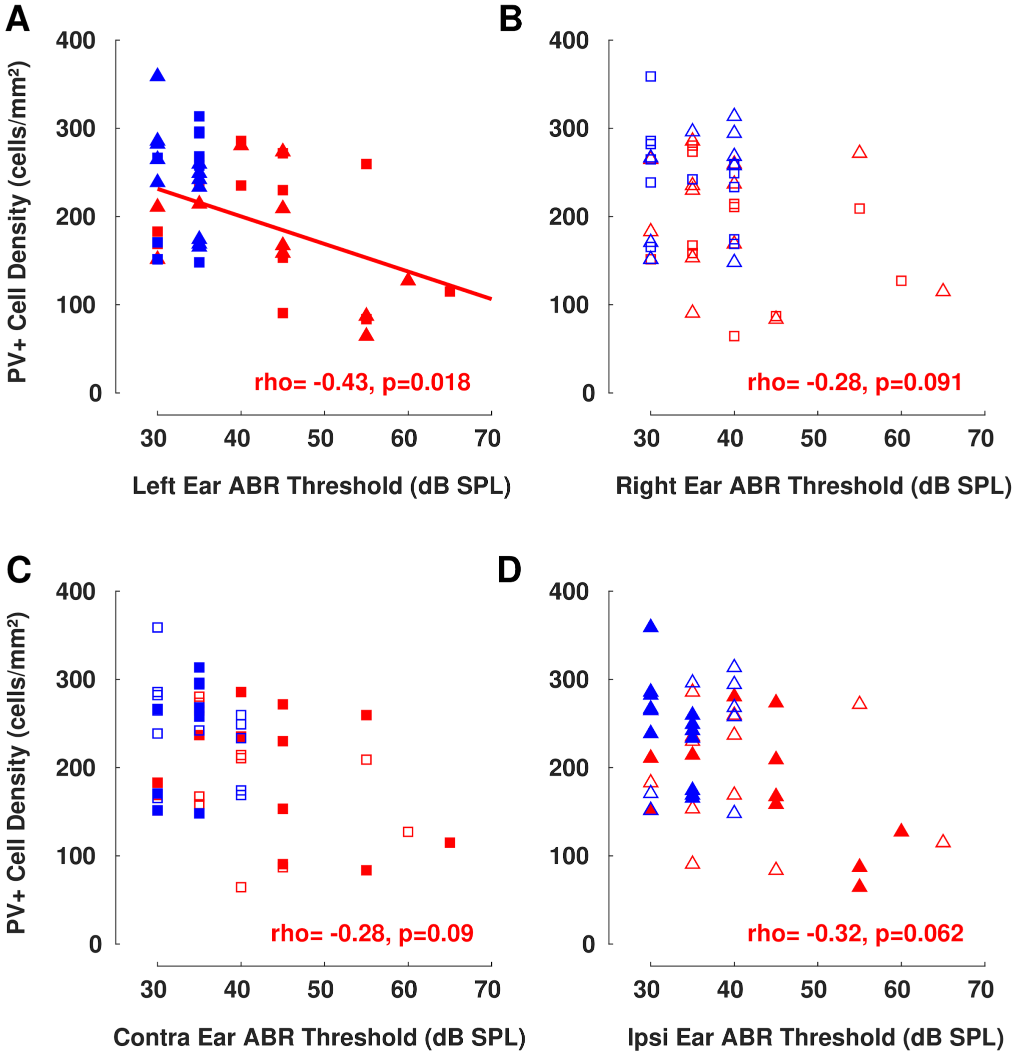

Figure S3. Correlations between PV+ cell density in the auditory cortex and left, right, contralateral, or ipsilateral ear ABR thresholds in *Df1/+* mice.

Key to symbols: blue, WT; red, *Df1/+*; filled, left ear; open, right ear; squares, contralateral ear; triangles, ipsilateral ear. Red text, Spearman's rho and p-value for correlation of PV+ cell density with specified ear ABR threshold, for *Df1/+* mice only.

(A) PV+ cell density in the auditory cortex of *Df1/+* mice shows a significant negative correlation with click-evoked ABR threshold in the left ear. Solid red line, two-dimensional least-squares linear fits to the *Df1/+* data.

(B-D) No significant correlations but similar trend to inverse relationship ($0.05<p<0.1$) between PV+ cell density in the auditory cortex of *Df1/+* mice and ABR thresholds in the right ear (B), contralateral ear (C), and ipsilateral ear (D).

Numbers of hemispheres and mice as in Table 1 and Figure 6.

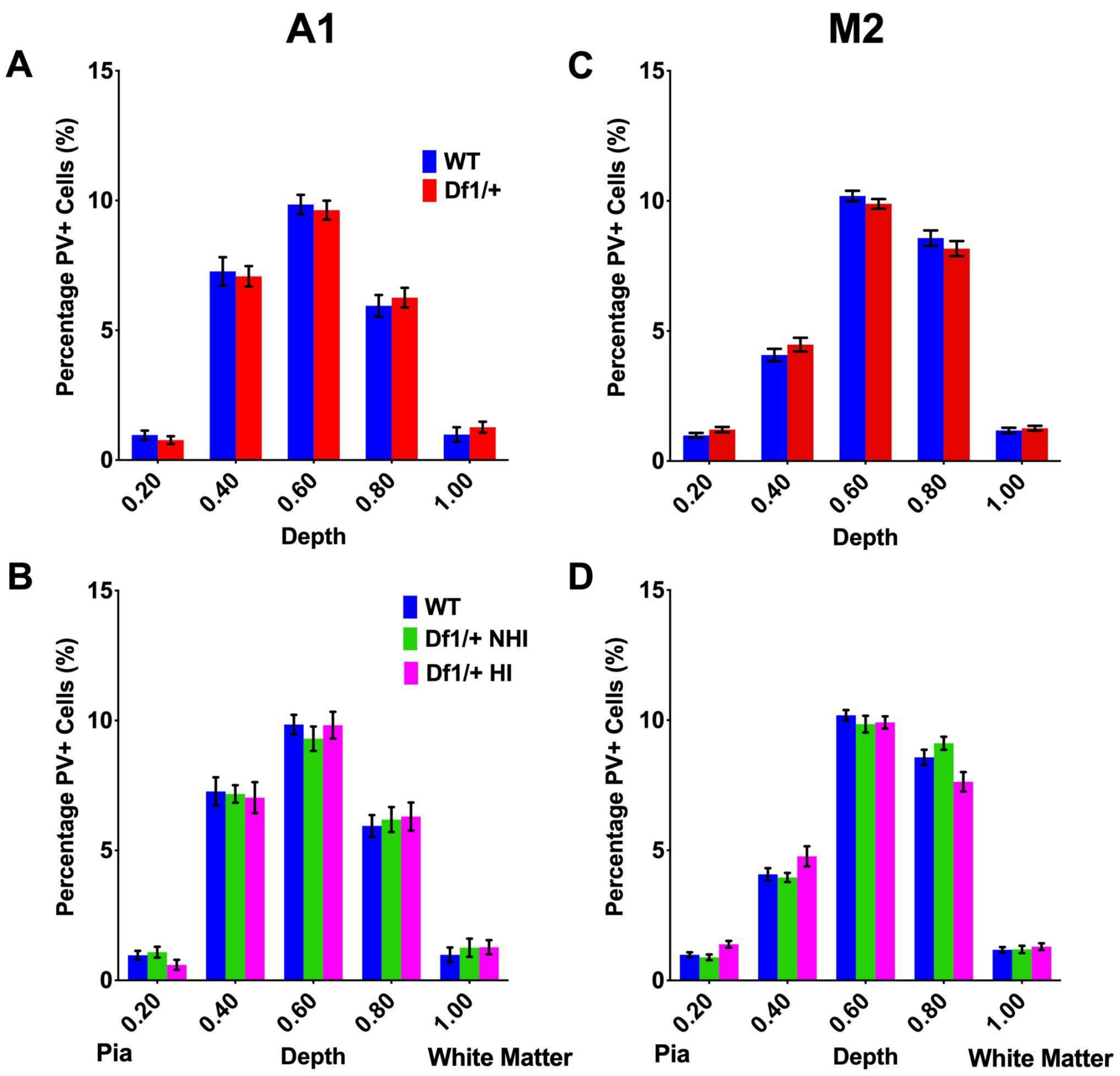

Figure S4. Minimal effect of *Df1/+* genotype or hearing impairment on laminar distribution of PV+ interneurons, in either auditory cortex or motor cortex. Depth distribution of PV+ cells was analyzed in 5 bins representing equal proportional depths in the cortex from pia to white matter. Error bars indicate SEM across hemispheres; see Table 1 for number of hemispheres and mice.

(A-B) PV+ cell depth distribution in A1, compared between WT and *Df1/+* mice (A) or between WT mice, *Df1/+* mice without hearing impairment, and *Df1/+* mice with hearing impairment (B). Two-way ANOVA

revealed no significant interactions between genotype and bin depth (WT vs. *Df1/+,* F(4,164)=0.25, p=0.91; WT vs. *Df1/+* NHI vs. *Df1/+* HI, F(8,160)=0.22, p=0.99).

(C-D) PV+ cell depth distribution in M2, compared between WT and *Df1/+* mice (C) or between WT mice and *Df1/+* mice with or without hearing impairment (D). Two-way ANOVA revealed no significant interaction between genotype and bin depth for WT vs. *Df1/+* mice (F(4,212)=1.13, p=0.34), but a weak interaction for WT vs. *Df1/+* NHI vs. *Df1/+* HI mice (F(8,208)=2.24, p=0.026). Post-hoc Tukey's multiple comparisons test identified the significant difference in M2 as arising from the 0.8 bin, corresponding to depths 0.6-0.8 of the total distance from pia to white matter (WT vs. *Df1/+* HI p=0.011, *Df1/+* NHI vs. *Df1/+* HI p=0.0022). For analyses performed with 10 or 20 equal-proportion depth bins (data not shown), there was no significant interaction between genotype and bin depth nor any single bin with significant difference between groups (all comparisons p>0.05).

SUPPLEMENTARY METHODS AND MATERIALS

Animal breeding and maintenance

Experiments were conducted in *Df1*/+ mice (also known as Df(16)1/+ mice) and their wild-type (WT) littermates bred from a genetically modified line established previously on the C57BL/6J background (1). Breeding was maintained by crossing *Df1*/+ males from within the colony with WT C57BL/6J females either from within the colony or from local vendors (Charles River UK; Jackson Labs USA). Back-crossing into the C57BL/6J strain had been maintained for well over 10 generations at the start of the experiments. Animals used in most of the experiments were bred and maintained at University College London and tested at the UCL Ear Institute. An additional cohort of *Df1/+* and WT mice derived from that colony was maintained at the National Institute of Mental Health and used for longitudinal tests of hearing ability in the Mouse Auditory Testing Core Facility of the National Institute on Deafness and Other Communication Disorders. All mice were maintained on a 12h on, 12h off light/dark cycle.

Auditory brainstem response and auditory evoked potential recording procedures

To screen mice for hearing impairment we recorded the auditory brainstem response (ABR), an electroencephalographic signal measured through the use of scalp electrodes which allows for the detection and visualization of sound-evoked potentials generated by neuronal circuits in the ascending auditory pathway (2). Previous work has already demonstrated that elevated ABR thresholds in *Df1/+* mice correlate with histological evidence of middle ear inflammation (3).

The main cohort of mice used in this study underwent ABR testing at UCL in the UK. ABR recordings were performed in a sound isolation booth (Industrial Acoustics Company, Inc.). Auditory stimuli were generated at a sample rate of 195,312.5 Hz using a digital signal processor (Tucker-Davis Technologies, TDT RX6), attenuated as needed (TDT PA5), amplified (TDT SA1), and presented using a free-field speaker (TDT FF1) positioned 17-18 cm from the ear directed toward the speaker. Speaker output was calibrated to within ±2 dB of target values before each set of experiments using a Bruel & Kjaer ¼ inch microphone (4939), placed at the location of the ear to be tested. Data was acquired at a 24,414 samples/sec (TDT RX5) using a low-impedance headstage and signal amplifier (TDT RA4LI and RA16SD, 20x gain overall, 2.2 Hz - 7.5kHz 2nd-order bandpass filtering, 800 µs group delay) along with a custom low-pass filter designed to remove attenuation switching transients (100 kHz cutoff). Stimulus presentation and data acquisition was controlled using software from TDT (Brainware) and custom software written in MATLAB (Mathworks).

Mice were anaesthetized via intraperitoneal injection of a ketamine-medetomidine cocktail (0.003-0.01 ml/g body weight of 10mg/ml ketamine, 0.083 mg/ml metetomidine). Body temperature was maintained at 37-38°C using a homeothermic blanket (Harvard Apparatus). Subdermal needle electrodes (Rochester Medical) were typically inserted under the skin at the vertex (positive), at the bulla behind the ear directed toward the speaker (negative), and over the olfactory bulb (ground). In animals for which ABRs but not AEPs were recorded, we instead placed the ground electrode behind the bulla opposite the tested ear. ABR thresholds were determined in the left and right ears in turn in most animals. The animal was re-oriented between recordings to direct the ear being tested toward the speaker, but we avoided altering the positions of the subdermal electrodes by switching electrodes at the input of the pre-amplifier whenever possible. ABR stimuli were 50 µs monophasic clicks ranging in sound level from 0 to 90 dB SPL in 5 dB steps, repeated 500 times at an inter-click interval of 50 ms.

In a subset of the animals tested at UCL, we collected further ABR recordings in combination with auditory cortical evoked-potential (AEP) recordings. To measure AEP signals, additional subdermal electrodes were placed at locations corresponding approximately to the left and right auditory cortices, and referenced to the same ground (at the olfactory bulb) as for the ABR recordings in these animals. Stimuli were 80 dB SPL clicks presented 1000 times at an inter-click interval of 300 ms. The longer inter-click interval allowed for resolution of late cortical AEP waves as well as the earlier ABR signals.

An additional cohort of *Df1/+* and WT mice were maintained and underwent longitudinal ABR testing at the National Institutes of Health in the USA. Procedures for longitudinal ABR testing conducted at the NIMH/NIDCD were similar to those at UCL, with the following differences. Ketamine-dexmedetomidine cocktail was used rather than ketamine-medetomidine; the negative electrode was placed below the ear rather than at the bulla; and stimuli were delivered via an in-ear coupler rather than free-field. Body temperature was maintained with a World Precision Instruments homeothermic blanket (ATC-1000 or ATC-2000), and stimulus delivery and data collection were accomplished with Tucker-Davis Technologies RZ6 processor, MF-1 speakers, and BioSigRZ software. Stimuli were 5 µs clicks or 3 ms tones (8, 16, 32 and 40 kHz), averaged over 512 click or 1024 tone repetitions at an inter-stimulus interval of 50 ms. (Due to technical concerns about data collected during delivery of 32 and 40 kHz tones, we report longitudinal ABR results only for clicks and 8 or 16 kHz tones.) Sound level was increased from 0 dB to a maximum of 90 dB SPL in 5 dB steps to identify threshold. Finally, mice tested at NIMH/NIDCD were recovered from anaesthesia (with a subcutaneous injection of atipamezole) for repeated longitudinal ABR testing at weekly intervals when possible, rather than immediately terminated for histology as in the UCL experiments.

Histological and immunohistochemical processing

Mice used for immunohistochemistry were euthanized using an overdose of sodium pentobarbital (0.1-0.2 ml of 20 mg/ml Euthatal; Rhône Mérieux, Essex, UK) and perfused transcardially with at least 30 ml 4% paraformaldehyde (Merck, Dorset, UK) using a peristaltic pump. Brain tissue was removed and stored in 4% paraformaldehyde at 4°C until histology could be performed. Brains were transferred to increasing concentrations of sucrose solution (15% followed by 30%) for cryoprotection before freezing and slicing. Coronal sections 50 µm thick were cut using a cryostat (Bright Instruments).

Sections were then stained alternately for parvalbumin (PV) and Nissl substance (a cell nucleus marker), or triple-stained for PV, NeuN (a pan-neuronal marker) and DAPI (a cell nucleus marker). Nissl staining was performed on mounted sections with cresyl violet, using standard procedures. For PV and NeuN staining, sections were incubated with 0.5% Triton and blocked with 0.5% Triton and goat serum blocking solution before being incubated in mouse anti-PV monoclonal antibody (1:2000, Sigma-Aldrich P3088) and rabbit anti-NeuN monoclonal antibody with species reactivity for mouse (1:2000, Merck Millipore MABN140) at 4°C overnight. Sections were then rinsed in PBS and incubated with goat anti-mouse secondary antibody (1:200, Sigma-Aldrich T5393) and goat anti-rabbit secondary antibody (1:200, ThermoFisher Scientific A-11008) for one hour at room temperature on a shaker, then rinsed again in PBS. Slides were then mounted with DAPI Flouromount-G (Southernbiotech) and coverslipped.

SUPPLEMENTARY REFERENCES

1. Lindsay EA, Botta A, Jurecic V, Carattini-Rivera S, Cheah YC, Rosenblatt HM, et al. Congenital heart disease in mice deficient for the DiGeorge syndrome region. Nature. 1999 Sep 23;401(6751):379–83.
2. Willott JF. Measurement of the auditory brainstem response (ABR) to study auditory sensitivity in mice. Curr Protoc Neurosci. 2006 Feb;Chapter 8:Unit8.21B.
3. Fuchs JC, Zinnamon FA, Taylor RR, Ivins S, Scambler PJ, Forge A, et al. Hearing loss in a mouse model of 22q11.2 Deletion Syndrome. PLoS ONE. 2013 Nov 14;8(11):e80104.
